# Prioritizing adaptation and mitigation in the climate movement: evidence from a cross-national protest survey of the Global Climate Strike, 2019

**DOI:** 10.1007/s11027-022-10003-y

**Published:** 2022-07-25

**Authors:** Joost de Moor

**Affiliations:** Sciences Po, Center for European Studies and Comparative Politics (CEE), Paris, France

**Keywords:** Climate mitigation, Climate adaptation, Transformation, Climate movement, Climate justice

## Abstract

Climate adaptation is seen by many as increasingly important and as deeply political, leading some to argue for its democratization. Social movements could play an important role in this. Meanwhile, we have recently witnessed a major swell in climate activism, as well as a growing realization among climate activists that it may be too late to prevent major climate disruptions. Yet to what extent this may lead to a focus on adaptation in the climate movement remains understudied. To address this gap in the literature, the current paper draws on survey data from 2,344 participants in Fridays For Future climate demonstrations in September 2019 in 13 cities in Europe, Australia and the USA. The analyses show that while one-half of the respondents still attributes greater weight to mitigation, the other half attributes equal weight to adaptation and mitigation, indicating a greater emphasis on adaptation than previously assumed. It is found that those supporting (equal focus on) adaptation experience less hope about the effectiveness of climate policies, and portray a reluctance to support far-reaching climate action. The latter indicates that support for adaptation in the climate movement is associated with conservative attitudes, indicating constraints for the emergence of a climate movement for transformational adaptation.

## Introduction

Besides mitigation, adaptation is generally seen as the main response to climate change. While the former aims to prevent and reduce the impacts of climate change through measures like CO2 reductions, the latter considers how society should respond to actual or unavoidable climate impacts, such as flooding, drought, urban heat, and fires. As climate impacts become increasingly noticeable and locked-in across the world, adaptation has gained scholarly and political attention (Bassett and Fogelman 2013). Simultaneously, we see a swell in climate activism to demand that governments take stronger climate action, in particular through the recent efforts of the global Fridays For Future (FFF) campaign that followed Swedish activist Greta Thunberg’s famous school strike (de Moor et al. 2020b). However, these two observations have not yet been connected, thus overlooking to what extent mitigation and adaptation play a role in the demands of climate activists like those in FFF. Climate activism is typically assumed to be about mitigation (e.g. Brulle 2014; Roser-Renouf et al. 2014), without investigating what role adaptation plays. The current paper addresses this gap in the literature by analyzing attitudes towards mitigation and adaptation in the Global Climate Strike organized by FFF in September 2019.

There are various reasons to be concerned about climate activists’ engagement not just with mitigation but also with adaptation. Adaptation fits within the boundaries of climate change movements’ (CCM) demands for firmer government action and justice in the face of climate crisis, and it resonates with observations about an increasing concern among climate activists that it might be ‘too late’ to prevent major climate impacts (Stuart 2020; Cassegård and Thörn 2018). While the impacts of such concerns are often more contradictory (Friberg 2021; de Moor 2021), one observable effect is that some activists shift from trying to prevent impacts through climate mitigation to imagining what responses to those impacts could entail once they become accepted as unavoidable (e.g. Bendell 2018). (It is worth noting that this change particularly presents a recent phenomenon in the global North, as ‘frontline’ and indigenous communities have been forced to deal with actual climate impacts and other environmental disruptions for much longer (Whyte 2017)). There are furthermore normative arguments for the involvement of climate movements with adaptation, as critical adaptation scholars are increasingly putting forward arguments about its political nature (O’Brien 2012; Adger 2016), the concurrent need to democratize it ((Mikulewicz 2018; Schlosberg et al. 2017), and the role that social movements could play in that process (Schlosberg 2013). This situation clearly calls for a closer look at the role of adaptation in recent climate activism.

Focusing on the case of FFF, this paper therefore aims, firstly, to investigate whether among today’s climate activists we might find evidence that such engagement with adaptation is materializing. More concretely, it analyzes how important climate activists consider climate adaptation and mitigation to be when taking the streets to demand that governments take stronger climate action. The second aim of the paper is to explore why some climate activists attribute more weight to adaptation than others do. The paper draws on survey data from 2,344 participants in FFF’s September 2019 *Global Climate Strike* campaign in 13 cities in Europe, Australia and the USA. Taking place primarily but not exclusively in the global North, these events are seen as the largest global climate mobilization in history, bringing together experienced as well as novice climate activists (de Moor et al. 2020b). While certainly not representative of the highly diverse phenomenon of climate activism, the size, spread and diversity represented in these demonstrations provided a unique opportunity to analyze a broad cross-section of climate activists in the global North.

The results show that just under half the activists prioritize climate mitigation over adaptation. The majority attributes equal weight to adaptation and mitigation. While close to no one prioritized adaptation, these findings suggest a bigger emphasis on adaptation than is generally reflected in the literature on climate activism, which focuses on mitigation and has largely ignored adaptation. What sets apart those giving equal weight to adaptation and mitigation from those that prioritize the latter is, firstly, feeling less hope about the effectiveness of climate policies, and secondly, more conservative attitudes to climate action, including a reluctance to prioritize mitigation over economic growth, majoritarianism and social security. This has implications for the kinds of politics on adaptation we may expect to come from climate activists.

## Adaptation and mitigation as potential goals for the climate movement

With climate scientists agreeing that serious climate disruptions are already here or under way, while stressing that worse scenarios can and should still be prevented, a consensus has emerged about the need for both mitigation and adaptation in response to climate change (IPCC 2014). Many argue that the two should not just be addressed simultaneously, but that they can even be advanced with mutual benefits (IPCC 2014). For instance, cases of urban ‘greening’ can address flooding and heat island problems (adaptation) while drawing CO2 from the atmosphere (mitigation). However, neither adaptation nor mitigation can be achieved entirely through such synergetic interventions. This introduces an opportunity cost, as choices must thus be made regarding the attribution of limited resources. Such dilemmas are particularly salient where measures to mitigate or adapt are mutually detrimental. Seawalls, for instance, have considerable carbon footprints. Hence, while governments like to propagate win–win scenarios in response to climate change, these are available only sometimes.

Underlying imaginaries may present further tensions between mitigation and adaption (Moser 2014). In terms of temporality, mitigation requires a story that it is not yet too late to prevent ‘runaway climate change’, whereas adaptation emphasizes that (some) impacts of climate change can no longer be avoided. While these narratives are not unreconcilable, in practice, they often conflict because talking about adaptation is considered fatalistic (de Moor 2021). Furthermore, in terms of prognosis, mitigation demands radical social change, by shifting away from a fossil-fuel-based economy or even from the dominant economic growth paradigm (Jackson 2016), whereas mainstream approaches to adaptation have been depicted as a conservative project to increase the resilience of the status quo (Bassett and Fogelman 2013; Hodson and Marvin 2009). For instance, critics have pointed out that adaptation has often prioritized protecting prestigious projects and zones of economic importance over vulnerable communities (Hodson and Marvin 2017; Anguelovski et al. 2016). Climate activists in particular have furthermore described adaptation as a techno-optimistic excuse not to take the radical measures needed in order to mitigate climate change (Simonet and Fatorić 2016; Remling 2018; de Moor 2021).

While some have thus rejected adaptation because of its conservative connotations, recent discussions have increasingly focused on rethinking adaptation. Particularly, there is a growing academic movement articulating adaptation in political terms (e.g. Meerow and Mitchell 2017; Ribot 2011; Adger et al. 2009). According to Eriksen et al. (2015), we need to recognize that:

what counts as “adaptive” is always political and contested. What is seen as positive adaptation to one group of people may be seen as mal-adaptation to another, and political processes determine which view is considered more important at different scales and to different constituencies. (p. 523)

Thus, adaptation is not merely political because it involves contestations over the distribution of adaptive resources, but because what constitutes good adaptation is contested (Felli and Castree 2012). Drawing on the wider literature on the (often obscured) political nature of environmental politics (Kenis and Lievens 2014), adaptation is here thus understood as political because it always involves winners and losers—be that kept implicit or made explicit (Mikulewicz 2018). While mainstream discussions of adaptation (e.g. on behalf of governments) tend to focus on the objective necessity of certain interventions that are then framed in ‘neutral’ terms of managing risks through science and technology, critical scholars underline that adaptation is always a matter of prioritizing some options over others, and unless challenged, priorities will reflect vested interests (Anguelovski et al. 2016; Hodson and Marvin 2017).

Adding substance to calls for politicization, critical adaptation scholars furthermore propose ‘transformational’ views of adaptation. While some only understand ‘transformational’ to indicate measures that are far-reaching (Kates et al. 2012), others specify that such far-reaching measures should address questions of justice and equality, shifting focus to a more fundamental debate on rethinking society in the face of climate disruptions (Nightingale et al. 2020). For instance, Pelling’s definition of transformational adaptation as ‘concerned with the wider and less easily visible root causes of vulnerability’ (2011, 86) makes it clear that transformational adaptation is far-reaching in the sense of addressing fundamental political questions about the economic and political organization of society. Likewise, Schlosberg et al. (2017) have argued that:

Only an approach to adaptation that moves beyond a sole focus on the biophysical risks of climate change, to one that considers the larger and more complex processes that interact and produce vulnerability, can address social, environmental, and climate injustice. (p. 414).

In sum, adaptation cannot be politically neutral, may imply a far-reaching overhaul of society, and involves questions of justice. Several authors interpret this conclusion as a clear imperative to democratize adaptation (Mikulewicz 2018; Burnell 2012). They echo advocates of the ‘enabling approach’ to sustainability transformations (Scoones et al. 2020) who have argued that adaptation should involve communities who know best for themselves what they need to thrive in the face of climate change. Similarly, Schlosberg et al. (2017) advocate for a capabilities approach to just adaptation that starts from the needs of communities to inform interventions needed to support them. However, Mikulewicz (2018) emphasizes that democratization cannot be reduced to the ‘involvement’ of communities through participatory processes or ‘community-based adaptation’ (CBA) approaches. The former have often been found to be tokenistic processes to legitimize pre-determined outcomes, and while the latter may imply more meaningful engagement, they risk shifting responsibilities—not power—to communities (Few et al. 2007). Ultimately, democratization has to be mainly about identifying and redressing power imbalances that underlie adaptation-based injustices (Mikulewicz 2018): it is the disempowerment of certain communities that renders them vulnerable to maladaptation. Democratization is then understood as being about redressing maladaptation through empowerment that allows vulnerable communities to politicize and challenge mainstream adaptation.

### What role for the climate movement?

While there is no simple answer to how adaptation should or could be democratized and politicized, the shift in power that is described above has, historically, often involved social movements (Rossi and della Porta 2015). While social movements may not be a sufficient condition when it comes to redistributing power, they could present necessary conditions for such change (Amenta et al. 2019). In the field of sustainability transformations in particular, political outsiders like social movements are seen as key to enabling transformations because compared to political insiders they are relatively well-positioned to challenge vested interests (O’Brien 2012; Temper et al. 2018). Social movements—and climate change movements (CCMs) in particular—may thus be important and even necessary conditions for the politicization and democratization of adaptation. Crucially though, whether CCMs take up this role has hardly been studied (Zografos et al. 2020; Boda and Jerneck 2019).

The CCM has been defined as ‘a loose, but nonetheless highly active umbrella structure which is supported, shaped and used by a multiplicity of civil society actors who are active in climate politics’ (Dietz and Garrelts 2014, 7). This definition captures the fact that the CCM is a highly diverse and loosely connected field of actors, which has over time been internally divided over conflicts regarding, among others, reformism and radicalism (Saunders 2012), climate justice (Hadden 2015) and the movement’s relation to governments (de Moor 2018). It is thus arguably preferable to refer to CCMs in plural, and it is only possible to assess the potential contribution of part of the CCM within the scope of this paper. By studying the FFF campaign in Australia, Europe and the USA, this paper focuses on a CCM based in the global North and a part of the movement that has been depicted as politically and strategically moderate (Marquardt 2020; de Moor et al. 2020b; Buzogány and Scherhaufer 2022). References to climate activism below are made with this focus in mind. Nonetheless, especially during its largest global campaigns, such as the one covered here, FFF mobilized protesters who reflected the movement’s strategic and political broadness (de Moor et al. 2020b).

CCMs have long been assumed to be focused on mitigation (e.g. through actions to stop fossil-fuel use and extraction) and to be skeptical towards adaptation for reasons discussed above (Brulle 2014; Dietz and Garrelts 2014; de Moor 2021). Still, CCMs are not unlikely to have or adopt a focus on adaptation. For instance, the emerging literature on postapocalyptic environmentalism (Cassegård and Thörn 2018) suggests that CCMs are increasingly convinced that a major apocalypse can no longer be prevented, thus raising the need to adapt to climate disruptions on the agenda of Northern CCMs.

Concurrently, Schlosberg has claimed that ‘movements are turning increasingly to adaptive responses to a changing climate – addressing, for example, urban heat, food security, or mobility’ (2013, 47). Case studies show that in particular cases, movements indeed become strongly engaged with adaptation-related topics. In particular in the USA, studies show that in the aftermath of hurricanes, social movements have organized to demand more just responses to climate induced disasters (Dawson 2019; e.g. Bullard and Wright 2009). Moreover, groups like Dark Mountain and Deep Adaptation advocate a shift in focus towards dealing with, rather than preventing, societal collapse. Nonetheless, these groups have also remained fairly marginal within the CCM, and research on a broader range of European CCMs has shown a considerable reluctance to engage with adaptation (de Moor 2021). Furthermore, existing, typically qualitative research, has focused on movement leaders, and it has consequently remained unclear how climate activists more generally relate to adaptation. Expectations contradict, and beyond case studies, hardly any research has systematically analyzed to what extent adaptation does or does not play a role in today’s CCMs.

The first aim of this paper is therefore to address this gap in the literature by interrogating to what extent climate activists actually perceive measures to adapt to climate change as an important goal when taking the streets. In particular, given that recent campaigns like FFF have focused especially on demanding government action on climate change (de Moor et al. 2020b), the paper will look into whether protesters in these campaigns believe that such government action should prioritize mitigation, prioritize adaptation or address both equally. Doing so will give us crucial insights into whether these climate activists are still by and large focused on mitigation, as has often been assumed, or whether adaptation may be taking up a place of greater importance. If the latter is the case, a presumable precondition for FFF to play a role in democratizing and politicizing adaptation would appear to be fulfilled.

### Explaining climate activists’ attitudes on mitigation and adaptation

The second aim of this paper is to explain why some climate activists may prioritize mitigation over adaptation or vice versa. Based on previous studies, two potential explanations will be explored.

First, a focus on adaptation has been associated with a loss of hope regarding the possibility that climate mitigation can still prevent dangerous climate change (Simonet and Fatorić 2016; Cassegård and Thörn 2018). Similarly, Roser-Renouf et al. (2014) argue that a strong sense of political efficacy is an important precondition for activism for mitigation: the more one believes that mitigation can be effective, the more likely one is to (feel motivated to) join the action. However, they assume that support for adaptation should be unrelated to efficacy. By contrast, the current paper expects that climate activists who are less hopeful about the efficacy of climate action will be inclined to support a focus on adaptation.

It is important to stress that the relation between hopefulness and climate action is complex and context dependent. Kleres and Wettergren (2017) argue for instance that in Northern CCMs, emotions like fear and hopelessness are seen as unproductive and that ‘feeling rules’ therefore keep them out of strategizing. By contrast, Cassegård and Thörn (2018) emphasize that postapocalyptic narratives may sound defeatists at first, but are seen by some to produce hope through the acceptance of loss and the imagination of what is possible after the apocalypse. Following this argument, adaptation can be seen as an ‘optimistic’ project. Nonetheless, those who feel more hopeless about the effectiveness of climate action will presumably consider greater impacts of climate change to be likely and may therefore attribute greater weight to adaptation. By contrast, those who feel more positive in this regard may feel that major impacts of climate change can still be prevented and will therefore (continue to) prioritize mitigation (Roser-Renouf et al. 2014). The first hypothesis this paper will test is therefore that:

- *H1: Hopelessness is related to greater support for climate adaptation.*

Second, attitudes towards adaptation can be expected to reflect activists’ more general attitudes on climate action. Specifically, a prioritization of adaptation is presumably related to more conservative views on climate action and a reluctance to support far-reaching, transformational climate action. Research suggests that the transformational views of adaptation discussed above have remained marginal compared to more conservative, mainstream approaches (cf. Simonet and Fatorić 2016; Bassett and Fogelman 2013). Research among climate movement leaders in particular suggests that this pattern is reflected as adaptation is often still understood as a conservative project—exceptions notwithstanding (de Moor 2021). If transformational views on adaptation are rare even among movement leaders, who can be expected to have more specialized knowledge of climate politics, it is to be assumed that such views would be even more rare in the rank and file of the movement.

Hence, adaptation is presumably still understood in mainstream terms and support for it would therefore be related to more conservative attitudes on climate action in general. More precisely, it can be expected that differences in the relative importance attributed to adaptation compared to mitigation follow a more general fold line within the climate movement, namely between those who are willing to change society radically and at considerable cost to mitigate climate change and those with less outspoken views on the matter (Marquardt 2020; Garrelts and Dietz 2014), who are less prepared to pay such costs, and who may be more prepared to accept the need to adapt to certain climate disruptions. In the case of FFF specifically, it has been found that some activists are willing to put climate action before economic growth, the will of the majority, and social security arrangements, while others are clearly more hesitant to accept such costs (Wahlström et al. 2019; de Moor et al. 2020a). It is thus hypothesized that those belonging to the latter group will attribute greater weight to adaptation to address climate change. By the same token, those more supportive of far-reaching climate action will be less supportive towards a focus on adaptation, instead preferring a focus on mitigation.

- *H2: A reluctance to support far-reaching climate action is related to greater support for climate adaptation.*

## Case selection, methods, data and measurements

This paper has sought to tap into a broad cross-section of climate activists from the global North to get a broad image of the role of adaption in CCMs today. Protest survey data were therefore used from demonstrations in 13 cities in the global North during the September 2019 Global Climate Strike, organized by FFF. The campaign was famously started by Swedish climate activist Greta Thunberg, who started her campaign in August 2018, when she decided to quit going to school and instead sat down in front of the Swedish parliament to demand appropriate climate action. Her actions drew widespread attention and support and soon escalated into a national and then global campaign of climate strikers. While initially targeted at fellow school students, the campaign over time grew substantially and increasingly attracted participants from all generations (de Moor et al. 2020a). In particular, the September 2019 Global Climate Strike week of action between 20 and 27 September was explicitly targeted at getting adults involved as well, for instance by encouraging trade unions to mobilize their members to join. Moreover, the strikes explicitly refrained from making any particular official demands, apart from insisting that governments take stronger climate action. Hence, in principle, the demonstrations offered a platform for demands for mitigation as well as adaptation. The September strikes became the largest climate mobilization in history. Thus, while we do not know how the protesters related to the wider population of climate activists, the strikes’ size, popularity and growing internal diversity made for a unique opportunity to analyze a broad cross-section of Northern climate activists’ characteristics and views. Still, not all climate activists participated or even supported the events and demonstrations are but the most visible moments of social movement activity. Any generalization of the findings should therefore be made with appropriate caution.

To get a representative sample of participants in these demonstrations, an international team of researchers implemented the well-established protest survey method developed by Van Aelst and Walgrave (2001). As described in detail elsewhere (de Moor et al. 2020a), the data collection followed the main principles outlined in this approach which makes sure that all protesters have an equal chance of being selected, thus yielding a random and therefore representative sample. In each surveyed demonstration, research teams handed out ~ 1,000 personal invitations to an online survey that interviewees were asked to complete after the demonstration. Two principles were followed to randomly select protesters. First, interviewers would not select interviewees themselves, as this could lead to selection bias based on interviewers’ perception of ‘approachable’ looking individuals (Walgrave and Verhulst 2011). Instead, interviewers would be sent to interview individuals who were selected by ‘pointers’ who followed the second principle. That is, pointers would apply a systematic protocol to make sure every protester had an equal chance of being interviewed. For instance, in a demonstration of approximately 10,000 protesters, every 10th individual would be selected. Finally, it was made possible to assess whether no biases occurred in the responses ultimately received from those who were selected to participate. Specifically, every fifth interviewee also received a short face-to-face interview that recorded basic characteristics, such as gender, age, education, political interest and protest experience. Since the response rate on these interviews tends to be higher than 90%, it offers a baseline to assess and potentially correct response biases. Based on this survey, the current paper presents survey data from 2,344 protesters in 13 cities across Australia (Sydney), the USA (New York) and all regions of Europe (Brussels, Vienna, Bern, Berlin, Chemnitz, Malmö, Gothenburg, Stockholm, Helsinki, Bucharest, Prague). Cities were selected based on the presence of a local research team that could carry out the survey. While thus presenting a convenience sample, our study includes several of the largest demonstrations from the September strike, thus contributing to the aim of obtaining a broad sample of climate activists. Each country team followed the ethical standards valid in their country or institution (including regarding informed consents and interviewing minors).

### Dependent variable

As mentioned above, the demonstrations were intended to put pressure on governments to take stronger climate action. Correspondingly, to tap into views on mitigation and adaptation, we asked respondents whether they thought the government action they were demanding should prioritize mitigation, adaptation or treat both equally. Specifically, they were asked:

- *If forced to prioritize the allocation of limited resources, what do you think the government in your country should do?*
  - *The government should prioritize measures to reduce climate change, such as lowering CO2 emissions.*
  - *The government should prioritize protecting people against the impacts of climate change, such as flooding, drought and forest fires.*
  - *The government should give equal priority to both.*
  - *Don’t know, no opinion.*

As discussed, there can be synergies between adaptation and mitigation in specific cases, but the question was designed to make respondents express a more general preference for mitigation or adaptation given limited resources for climate action. Thus, while the third option was included to allow respondents to indicate that both were equally important for them, the emphasis on prioritization and limited resources made it clear that this option did not imply that governments would spend more resources on climate action in general. Furthermore, to prevent forcing individuals to express preferences they might not have, the ‘don’t know/no opinion’ option was included.

As to the wording of the options, we avoided using the rather technical terms mitigation and adaptation, as this could limit understandability. Instead, the distinction was made concrete by referring to goals that are most commonly associated with mitigation and adaptation (reducing climate change through CO2 reduction versus protection from flooding, drought and forest fires). This was particularly important since many of the participants in the Global Climate Strikes were experiencing their first engagement with climate politics (de Moor, de Vydt, et al. 2020). The items therefore also did not specify the means by which these goals should be achieved—e.g. through conservative or transformational measures. While specifying this may have been relevant from a theoretical point of view, doing so would have risked reducing the understandability of the survey question. Furthermore, the immense data collection effort yielding the current number of interviews was only possible through a large international collaborative effort, which meant that the space available in our survey for each team involved was limited. Surveys that are too long reduce data quality significantly (Dillman et al. 2014). Hence, we could only dedicate space for one question to measure support for mitigation and adaptation.

### Independent and control variables

To measure hopefulness in relation to climate change (H1), respondents were asked to indicate on a 5-point scale ranging from ‘not at all’ to ‘very much’ to what extent they agreed with the two statements that ‘I feel hopeful about policies being able to address climate change’ and ‘Even if things look bleak, I do not lose hope that we are able to deal with climate change’. While the first item specifies the role of policymaking, which is important in a demonstration demanding climate policy, the second item measures a more diffuse sense of hopefulness. The correlation between both items is moderate (0.39, p 0.000) so including both separately in the analyses is preferable.

Support for far-reaching, transformational climate action (H2) involves various dimensions. After all, climate politics itself is a multidimensional issue. The FFF protest survey included three items that cover key aspects of debates about how far-reaching climate action should be. Respondents were asked to indicate to what extent they agreed or disagreed with the following statements (using a 5-point scale): ‘The Government must act on what climate scientists say even if the majority of people are opposed’, ‘Protecting the environment should be given priority, even if it causes slower economic growth and some loss of jobs’ and ‘Measures to decrease CO^2^ emissions cannot be allowed to make social welfare arrangements worse’. In short, these items measure whether respondents believe climate action should be allowed to trump basic principles of majoritarian democracy, economic growth and social security, respectively. Admittedly, these statements cannot be directly linked to any particular view on transformational adaptation, and it is instead the general support for far-reaching climate action that is implied in transformational approaches that is primarily tapped into. Scores for the latter item were reversed to make sure that higher scores on all items indicate greater support for far-reaching measures. The correlation between these items is moderate (0.41, p 0.000) between the first two items, non-significant between the first and third and weak between the second and third (0.07, p 0.001). It is therefore again preferable to include them separately in the analyses.

Following previous research on political attitudes among protesters and in the climate movement more specifically (e.g. Emilsson et al. 2020; Wahlström et al. 2013; Wahlström et al. 2019), the analyses control for basic personal characteristics including age, gender, education and left–right placement. Age is particularly important considering the youthfulness of FFF and was accordingly recoded to distinguish those below the age of 20 from those above. Education was firstly coded following the international ISCED categorization and then recoded to ‘low’, ‘middle’ and ‘high’. Left–right self-placement was measured on a scale ranging from 0 (left) to 10 (right). Unsurprisingly, the participants overwhelmingly placed themselves to the left. To capture meaningful differences, the variable was therefore recoded into ‘far left’ (0–2), ‘centre left’ (3–5), ‘right’ (6–10), and a category for those who indicated that they did not know or found the distinction meaningless. Furthermore, previous experience with environmental or climate protest was included, as experienced activists may over time develop different beliefs regarding for instance the likely successfulness of mitigation. Political efficacy, or the belief that one can make a difference in politics, is also included to control for the possibility that a more general sense of fatalism (the opposite of efficacy) leads to a preference for adaptation. This item was measured with a rescaled sum-variable of the following three statements that respondents rated from ‘strongly disagree’ (1) to ‘strongly agree’ (5): ‘My participation can have an impact on public policy in this country’; ‘Organized groups of citizens can have a lot of impact on public policies in this country’; ‘If citizens from different countries join forces, they can have a lot of impact on international politics’. All items load more than 0.59 on the same factor in an exploratory factor analysis, and the resulting factor has high reliability (Cronbach’s alpha = 0.76). Finally, a dummy variable was added to distinguish European and non-European demonstrations. While the European cities in our study have only been exposed to relatively mild climate impacts, inhabitants of New York and Sydney have been exposed to more serious climate-related impacts like hurricanes (e.g. Hurricane Sandy, 2012) and forest fires (e.g. the widespread bushfires in 2019–2020). Such immediacy may have affected attitudes towards adaptation.

## Results

### Descriptive analyses

By presenting descriptive statistics on the control variables, Table 1 provides basic information on the protesters we included in our survey. They show that respondents included an unusual number of teenagers, confirming the popular image of FFF as a young movement. However, many more were older, suggesting that the September 2019 global wave of protest succeeded in broadening the movement in terms of age. There were considerably more women than men and participants were typically of a higher education background. Many were experiencing their first ever climate protest, but most had participated at least once before. Unsurprisingly, the protesters generally had a left-wing political leaning. In short, the September 2019 Global Climate Strike mobilized a highly diverse constituency, but a picture emerges as well of a typical protester as young, female and from a high education background with limited climate protest experience and a left-wing political orientation.

**Table 1:** Descriptive statistics of control variables

	Total (*N*)	Total (%)
Age (categorized)		
Under 20 years	561	34
Over 20 years	1.783	76
Gender		
Male	874	40.05
Female	1269	58.16
Other	39	1.79
Education		
Low	198	9.25
Middle	535	25.00
High	1407	65.75
Climate protest experience		
Never	640	29
1–5	1.201	55
6–10	224	10
11–20	73	3
21 +	45	2
Left–right self-placement		
Far left	1.016	47
Centre left	685	32
Right	123	6
No-opinion/meaningless	338	16

Before moving to describing our dependent and independent variables, it is worth mentioning that the data confirm the popular image that these demonstrations were strongly focused on demanding government action on climate change. No less than 91 percent of the respondents regarded their participation as a way ‘to pressure politicians to make things change’. It therefore makes sense, from the point of view of the respondents, to explore support for mitigation and adaptation in terms of what the government’s climate policy should focus on.

Table 2 gives an overview of the answers to the question what government policy should focus on. The fact that less than 2 percent of the respondents used the ‘Don’t know, no opinion’ option indicates that the question was meaningful to most respondents. Furthermore, we see that only 2 percent of the interviewees indicated that government should prioritize adaptation over mitigation. Support for a focus on mitigation was clearly much larger, with 45 percent. However, the largest group consisted of those individuals who believe that government should not prioritize either mitigation or adaptation in the distribution of limited resources (51%). While further research would be needed to flesh out climate activists’ attitudes on specific mitigation and adaptation policies, this finding challenges the common assumption that climate activism is primarily about mitigation. More than half of the respondents do not seem to share authors’ common preoccupation with mitigation.

**Table 2:** Distribution of preferences for prioritization of mitigation and adaptation, given limited resources

	*N*	%
Prioritize mitigation	990	45.25
Prioritize adaptation	50	2.29
Give equal priority	1112	50.82
Don’t know/no opinion	36	1.65

As to the independent variables, Table 3 shows that protesters varied considerably regarding their hopefulness in the face of climate change, with hope about policies’ ability to address climate change being somewhat lower than the more general measure of hopefulness. Overall, these findings are in line with previous studies showing that climate activists—despite dealing with humanity’s greatest challenge and limited progress or time to act—score fairly high on hopefulness (Wahlström et al. 2019). Less than 25% of respondents indicate feeling hopelessness. Nonetheless, hope is a complex, multidimensional emotion and fully understanding it demands a more elaborate analysis than is possible within the scope of this paper.

**Table 3:** Descriptive statistics of hope

	*I feel hopeful about policies being able to address climate change*	*Even if things look bleak, I do not lose hope that we are able to deal with climate change*
1. Strongly disagree (%)	6.06	1.78
2. Disagree (%)	18.63	11.03
3. Neither disagree nor agree (%)	32.71	30.86
4. Agree (%)	27.43	38.24
5. Strongly agree (%)	15.17	18.09
Avg	3.27	3.60
*N*	2195	2194

Table 4 gives an overview of levels of agreement with the items measuring support for far-reaching, transformational climate action. On the one hand, we see widespread support for the notions that government should do what climate scientists say even if the majority of people is opposed (‘Climate vs Majority’ avg. = 4.21/1–5), and that protecting the environment should be given priority, even if it causes slower economic growth and some loss of jobs (‘Climate vs Growth’ avg. = 4.53/1–5). Hence, there generally appears to be strong support for far-reaching measures to tackle climate change, though a smaller percentage of respondents did clearly indicate reservations to such change. There is bigger disagreement as to whether measures to decrease CO^2^ emissions should be allowed to make social welfare arrangements worse (‘Climate vs Social security’ avg. = 3.11/1–5), which may reflect the general support for—and reluctance to compromise—social security in progressive movements and particularly support for a ‘just transition’.

**Table 4:** Descriptive statistics of support for far-reaching climate action

	*The government should do what climate scientists say even if the majority of people is opposed*	*Protecting the environment should be given priority, even if it causes slower economic growth and some loss of jobs*	*Measures to decrease CO2 emissions are to be allowed to make social welfare arrangements worse*
1. Strongly disagree (%)	.64	.78	10.60
2. Disagree (%)	4.02	1.01	22.52
3. Neither disagree nor agree (%)	13.11	5.62	25.97
4. Agree (%)	38.42	29.31	26.92
5. Strongly agree (%)	43.81	63.28	14.00
Avg	4.21	4.53	3.11
*N*	2189	2187	2114

### Explanatory analysis

Because very few respondents believe that adaptation should be prioritized, the main difference to explain appears to be between those who wish to prioritize mitigation and those who do not. Therefore, respondents supporting prioritization of adaptation or of both equally are added to the same group. The latter group represents the positive cases (1) in a dichotomous variable where support for mitigation is negative (0). A logistic regression is modelled to explain the difference between belonging to the two groups. As a robustness measure, the analyses control for the clustering of standard errors within individual demonstrations (particular demonstrations may each have had some distinct characteristics that need to be accounted for). Moreover, the models have been subjected to standard statistical robustness checks, including multicollinearity, goodness-of-fit and model specification errors (UCLA: Statistical Consulting Group 2020).

The results in Table 5 paint a clear picture of what explains support for a focus of government action on adaptation and mitigation. First, as to the control variables, we see that men are less likely to support a focus on adaptation. Teenagers are marginally significantly more likely to prioritize mitigation, which may reflect an awareness that they will face considerable and potentially unsurmountable climate impacts within their lifetimes, thus inspiring greater tenacity and unwillingness to compromise on the goal of mitigation. However, this effect was not theorized and lacks sufficient significance to be considered meaningful for the current analysis. The effects of other control variables are not statistically significant.

**Table 5:** Logistic regression of support for prioritization of government action on mitigation (0) and for (equal) priority for adaptation (1)

Variable	Odds ratio	St. dev	*P*
Age (teenagers = 1)	.774	.116	.087
Gender (men = ref.)			
Women	1.449	.191	.005
Other gender identity	2.582	1.117	.028
Education (low = ref.)			
Middle	.828	.180	.384
High	.668	.158	.088
Left–right placement (far left = ref.)			
Centre left	1.013	.107	.902
Right	.738	.203	.269
Don’t know/meaningless	1.169	.131	.164
Climate protest experience	1.028	.039	.474
Political efficacy	.919	.065	.234
Non-EU (EU = ref.)	1.034	.196	.859
Hope (policy)	.906	.029	.002
Hope (general)	1.094	.084	.240
Climate vs Majority	.821	.034	.000
Climate vs Growth	.734	.060	.000
Climate vs Social security	.857	.047	.005
Intercept	26.351	15.467	.000
*N*		1957	
Pseudo R2		.04	
Log likelihood		− 1303.39	

In support of H1, we find that that hope about the ability of policies to address climate change is negatively related to support for (equal) priority for adaptation (OR = 0.906**). In other words, the more hopeful respondents are about the effectiveness of climate policies, the more likely they are to support a focus on mitigation. A one-unit increase on the 5-point scale of hope makes respondents roughly 10 percent less likely to support (equal) priority for adaptation. We do not find a similar effect for the more general sense of hope. This can be explained by the fact that the dependent variable is clearly about climate policy and will therefore be more likely to be influenced by a policy-specific measure of hope.

Next, we see that the model clearly supports H2: Support for far-reaching climate change action is negatively related to support for (equal) priority for adaptation. The effect is statistically significant for all three indicators. Thus, support for adaption is greater among individuals who oppose measures that go against the will of the majority of the people (OR = 0.827***), that slows economic growth or causes job loss (OR = 0.726***) and that could make social security arrangements worse (OR = 0.862*). By contrast, greater support for such radical measures is thus positively associated with greater support for a focus on mitigation in government’s climate policy. Thus, for the strongest effect, of support for measures that go against economic growth and causes job losses, a one-unit increase on the 5-point scale of agreement makes respondents roughly 23 percent less likely to support (equal) priority for adaptation.

An examination of the predicted probabilities of support for adaptation further helps us interpret these effects. The predicted probabilities presented in Table 6 show that each increase in the independent variables measuring support for far-reaching climate action gives a roughly equal increase in the probability that a respondent will support an (equal) prioritization of adaptation. In other words, we are looking at a linear relationship, whereby it is not one particular group of respondents (e.g. totally opposed to far-reaching climate action) that drive the effect. Rather, each step down the scale of support for far-reaching climate action leads to a roughly equal increase in support for adaptation.

**Table 6:** Predicted probabilities (PP) of support for (equal) priority for adaptation (with 95% confidence intervals (CI))

Value	Climate vs Majority			Climate vs Growth			Climate vs Social security		
	PP	CI		PP	CI		PP	CI	
1 Strongly disagree	.678	.613	.743	.769	.672	.866	.614	.520	.708
2 Disagree	.635	.575	.695	.711	.628	.795	.577	.499	.655
3 Neither	.590	.532	.648	.646	.579	.713	.539	.474	.604
4 Agree	.543	.483	.604	.575	.517	.633	.500	.439	.561
5 Strongly agree	.495	.428	.564	.500	.431	.570	.462	.394	.530

Predicted probabilities have been calculated based on the regression presented in Table 5 using the *Margins* comment in Stata 17

In short, those who support (equal) priority for adaptation are less hopeful about the efficacy of climate policies, and less willing to support far-reaching climate policies. These effects are quite large. While the pseudo R^2^ (0.04) indicates that the model does not explain a very large part of the variance, other conventional indicators do confirm that the model is well-specified.

## Discussion and conclusion

This paper has analyzed the role adaptation plays in climate activists’ demands. Using the case of the September 2019 Global Climate Strike organized by Fridays For Future, it was analyzed whether activists demanding government action on climate change prioritize mitigation, adaptation or both equally. The results show a fairly even split between those who want policies to prioritize mitigation and those who want them to focus on mitigation and adaptation equally. Very few respondents preferred a prioritization of adaptation. This finding suggests that adaptation plays a bigger role among climate activists than it is given credit for by the limited amount of attention this topic has received in the literature on climate activism. Alternative measures of support for mitigation and adaptation, including those assessing support for specific policies in specific places, should be explored to further explore and verify this picture. Yet tentatively, if more than half of climate activists in a large and inclusive mobilization like the 2019 Global Climate Strike attribute equal weight to both, the often implicit perception of the climate movement as primarily focused on mitigation (e.g. Brulle 2014; Dietz and Garrelts 2014)—such as through actions against fossil-fuel industry or demands for CO^2^ reduction—requires reconsideration.

This is arguably good news for critical scholars promoting the politicization and democratization of adaptation (Mikulewicz 2018; Meerow and Mitchell 2017; Adger 2016). While explicit and specific demands for adaptation have not been particularly visible in the demands of FFF (which have largely been kept general for strategic reasons (de Moor, de Vydt, et al. 2020)), many activists appear to demonstrate a willingness to put adaptation central in the movement’s demands, suggesting they would support movement leaders choosing to steer in this direction.

However, there is little evidence that this would necessarily extend to the more specific demands for transformational adaptation that many critical adaptation scholars see as the desired outcome of politicizing and democratizing adaptation. That is, it is precisely support for the kind of far-reaching changes that is advocated in transformational approaches that is negatively associated with support for adaptation among FFF activists: the more respondents oppose far-reaching climate action, the more likely they are to prioritize adaptation. More research is needed into the exact causal pathways and mechanism at work here, but a plausible interpretation is that support for adaptation originates from a reluctance to change society too radically to mitigate climate change, consequently leading to an acceptance of certain impacts as inevitable and a concurrent preparedness to adapt to them. The second half of this reasoning is at least supported by the significant relation that was found between a lack of hope in the effectiveness of climate policies and support for adaptation, which suggests that adaptation becomes a priority when, for whatever reason, mitigation is expected to fail.

While the findings presented in this paper thus suggest CCMs could be important actors in the politicization and democratization of adaptation, it remains unclear which actor(s) could be expected to take forward the cause of transformational adaptation. Surely, previously mentioned groups like Deep Adaptation are taking forward ideas that overlap with notions of transformational adaptation, yet its implications for the wider climate movement remain questionable. Potentially reflecting earlier findings suggesting that climate movement leaders often struggle to advance progressive notions of adaptation (de Moor 2021), adaptation seems to remain seen as, and associated with, conservative politics in the eyes of the climate movement’s rank and file. As Zografos et al. (2020) have recently noted, transformational adaptation, while attracting increasing academic attention, has not made the leap to actual politics. Boda and Jerneck warn that ‘calls for major structural transformations remain vague without identifying what viable agent and through which particular processes such changes can be accomplished’ (2019, 633). Transformational adaptation may be a desirable outcome of politicizing and democratizing adaptation, and the latter may present a necessary condition for the former, but more research is still needed to understand which actors may actually contribute to the realization of transformational adaptation and how (Carmin et al. 2016).

It remains important to emphasize that this paper covers only one mobilization, that the CCM is much broader than what is covered here and that case studies do demonstrate exceptions. Furthermore, since the collection of our data in 2019, the climate movement has been shaken up considerably by several events, thus demanding a reflection on the enduring relevance of these findings. Firstly, the Covid-19 pandemic and the social restrictions in response to it seem to have made somewhat of an end to the observed wave of climate mobilization (de Moor, de Vydt, et al. 2020). When the CCM reaches the next peak in its cycle of contention (Tarrow 2011), it will inevitably be in a changed form, introducing new actors, experiences, and politics—possibly also regarding adaptation.

Secondly, we have witnessed a further increase in extreme weather events in several of the countries included in this study, particularly in the summer of 2021, including historic floods in Belgium, Germany and the Netherlands, forest fires in the Mediterranean, storms and fires in the USA and unprecedented bushfires in Australia in 2019–2020. These events, alongside the IPCCs damning *Sixth Assessment Report* (2021) may increase the willingness to accept far-reaching climate action, which we found to be related to support for mitigation. At the same time, these events may decrease hopes that policies can still keep climate change within safe boundaries, which we found to relate positively to support for adaptation.

Ultimately, however, developments like these are not new. FFF emerged on the cusp of the summer of 2018 that was filled with extreme weather events as well, and just after the publication of an equally strong warning by the IPCC (its 2018 report on 1.5 degrees warming (2018)). Given those similarities, the impact of recent events may as well be limited, leaving the found association between adaptation, hopelessness and attitudes on far-reaching climate action intact. Future research will have to determine whether change or continuity prevails.

## Data Availability

Upon request.

## Code availability

Upon request.
